# Extraction, Isolation, Identification, and Characterization of Anthocyanin from Banana Inflorescence by Liquid Chromatography-Mass Spectroscopy and Its pH Sensitivity

**DOI:** 10.3390/biomimetics9110702

**Published:** 2024-11-15

**Authors:** Nuwanthi Senevirathna, Morteza Hassanpour, Ian O’Hara, Azharul Karim

**Affiliations:** 1 School of Mechanical, Medical and Process Engineering, Faculty of Engineering, Queensland University of Technology, Brisbane 4000, Australia; nuwanthi.senevirathna@hdr.qut.edu.au (N.S.); m.hassanpour@qut.edu.au (M.H.); i.ohara@qut.edu.au (I.O.); 2 Centre for Agriculture and the Bioeconomy, Faculty of Science, Queensland University of Technology, Brisbane 4000, Australia; 3 ARC Centre of Excellence in Synthetic Biology, Queensland University of Technology, Brisbane 4000, Australia; 4 ARC Industrial Transformation Training Centre for Bioplastics and Biocomposites, Queensland University of Technology, Brisbane 4000, Australia

**Keywords:** anthocyanin, antioxidant, banana inflorescence, cyanidin, pH-sensitive

## Abstract

Anthocyanin is an important flavonoid with antioxidant, anticancer, and anti-inflammatory properties. This research investigates the anthocyanin content of Cavendish banana inflorescence, a by-product often discarded as agricultural waste. The study employs two drying methods, namely oven-drying and freeze-drying, followed by accelerated solvent extraction using acidified water and methanol. Liquid chromatography-mass spectroscopy (LC-MS) results confirm banana inflorescence as a rich source of anthocyanins. According to LC-MS analysis, freeze-dried banana inflorescence extracted in methanol at 80 °C exhibits the highest anthocyanin content (130.01 mg/100 g). This sample also demonstrates superior characteristics, including a chroma value of 40.02 ± 0.01, a redness value of 38.09 ± 0.16, 18.46 ± 0.02 °Brix, a total phenolic content of 42.5 ± 1.00 mg/g, expressed as gallic acid equivalents, and a total antioxidant activity of 71.33 ± 0.08% when assessed with the DPPH method. Furthermore, the study identifies the predominant anthocyanin as cyanidin, along with the presence of other anthocyanins such as delphinidin (Dp), malvidin (Mv), petunidin (Pt), pelargonidin (Pg), and peonidin (Pn). Interestingly, the extracted anthocyanins demonstrate pH sensitivity, changing from red to brown as pH increases. These findings highlight the potential of utilizing Cavendish banana inflorescence for anthocyanin extraction, offering sustainable waste valorization methods with promising applications in biomimetics and bioinspiration fields.

## 1. Introduction

Banana is the world’s highest-producing fruit crop, amounting to over 100 million tonnes of production annually. The increasing demand for bananas has led to a significant rise in global production, emphasizing their status as the most consumed fruit. The global banana production was reported as 136 million tonnes in 2022, with a cultivation land of 5,940,149 Ha [1]. In Australia, bananas are the most consumed fruit, with a production of 374,251 tons in 13,670 ha of land in 2022. Among these, Cavendish is the most grown commercial cultivar, which constitutes 97% of total production in Australia [2]. However, banana crop production generates massive quantities of by-products/waste such as stems, leaves, and inflorescence (flower).

Banana inflorescence is the edible product among these by-products. This inflorescence is also known as the banana flower, navel, heart, pendant, and bell in different countries and regions. This banana inflorescence has been used as a food as well as a medicinal product in countries such as Thailand, China, Sri Lanka, and India due to its nutritional and medicinal benefits [3].

Some studies have proven the medicinal properties, such as antioxidant activity, anticancer, antiglycemic, and antimicrobial within the banana inflorescence [3]. These properties are based on the chemical composition of banana inflorescence. The banana inflorescence has shown a rich profile of bioactive compounds, including phenolic acids, flavonoids, saponins, triterpenes, and anthocyanins.

**Figure 1:**
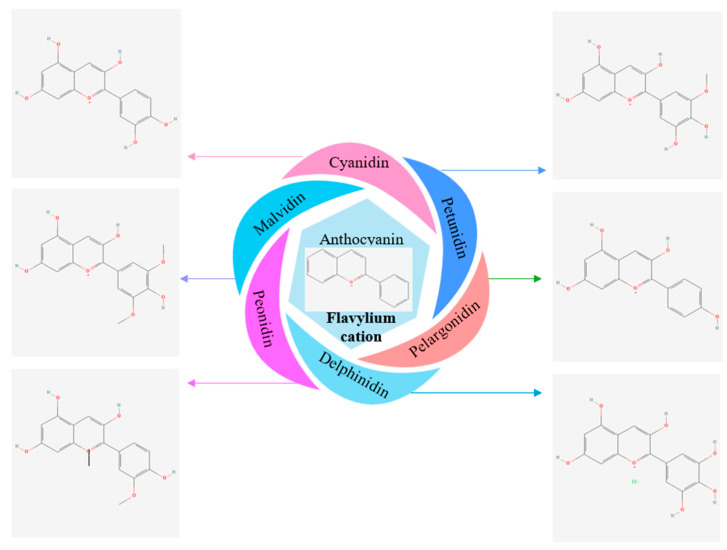
Major anthocyanin types found in nature.

Anthocyanins are polyphenolic natural pigment [4] flavonoids found in many vegetables and fruits, mainly berries [5]. Chemically, anthocyanin is a derivative of flavylium cation. As shown in Figure 1, more than 23 anthocyanins have been reported in natural products [6]. Anthocyanins have different colours, as they form different chemical structures according to the attachment of different radicals in R1 and R2, on the benzene ring. Naturally, six highly-abundant, chemically different anthocyanin types were identified [7]. They are cyanidin, delphinidin, malvidin, pelargonidin, peonidin, and petunidin [8,9]. The differences observed in the composition of anthocyanins can be attributed to a variety of factors that include environmental and growing conditions, genetic factors, as well as the timing of the harvest [10]. These factors can lead to variations in anthocyanin composition even within the same species, but from different geographic regions or cultivars [6].

Anthocyanin pigments are found in many plants, such as grapes, berries, cabbage, purple potatoes, black carrots, and hibiscus [11,12]. Anthocyanins are natural plant pigments which contribute to a range of colours in fruits, vegetables, and flowers. Chemically-different anthocyanin groups can be found in different colours such as red, orange, purple, violet, and blue. Anthocyanins are generally found in vacuoles and sub-epidermal cells within the plant tissues [13]. These compounds are pH-sensitive and water-soluble and have antioxidant activity [14,15,16]. These compounds are gaining high scientific attention, owing to their potential medicinal benefits [17,18] and the fact that they are sources of a synthetic colouring agent [19,20].

The colour-changing properties influenced by external factors such as pH and temperature, which can inspire designs for bio-compatible sensors in bionics. These colour characteristics could be applied to create biosensing materials or visual indicators for health diagnostics or environmental monitoring devices.

**Figure 2:**
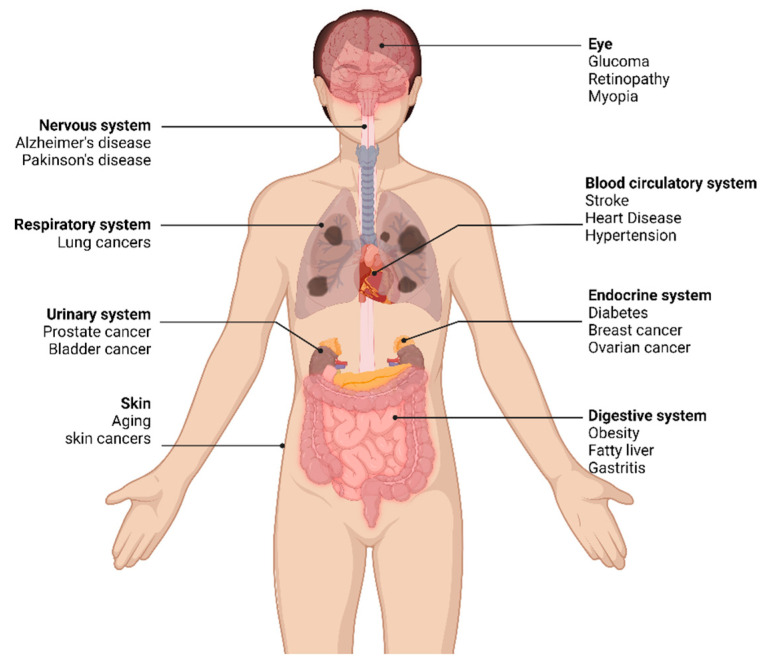
Medicinal benefits of anthocyanin.

Recent studies have shown that anthocyanins have potential health benefits, including anticancer, antimicrobial, cytoprotective, and antitumor activities [6]. Moreover, anthocyanins help in eliminating obesity and diabetes. The medicinal potential of anthocyanins is attributed to their antioxidant [21] and anti-inflammatory activities [22,23], as illustrated in Figure 2. The therapeutic potential of anthocyanin [24] suggests that anthocyanin-rich foods are becoming attractive for the food and biomedical industries [19,22]. Moreover, anthocyanin could be utilized as pure, biologically-inspired medicines, specifically for humans with allergic reactions to synthetic antioxidants. However, as these compounds are chemically bound to the cell vacuole, the first step of the production process is the efficient extraction of anthocyanins [25].

**Figure 3:**
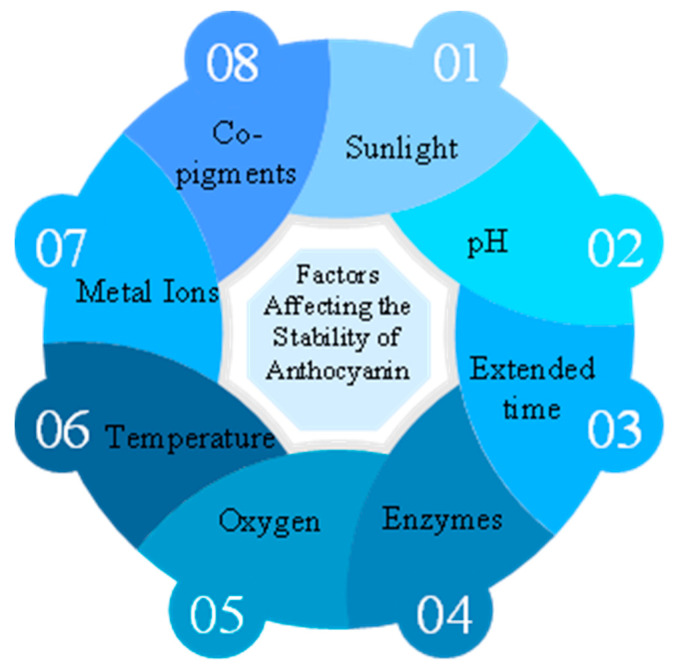
Factors affecting the stability of anthocyanin.

Previous studies have shown different extraction methods used for anthocyanin extraction from natural plants [26]. However, anthocyanins are highly unstable and subject to oxidation under the influence of temperature, oxygen, enzymes, water, co-pigments, metal ions, and pH [4], direct sunlight [5]. These barriers cause difficulties in the extraction process of anthocyanin. These influencing factors are summarized in Figure 3. Understanding the process of extracting anthocyanins can contribute to bio-fabrication techniques where natural plant pigments are used in biomedical industries.

As anthocyanins are highly unstable, extraction methods which require longer times and high exposure are not favourable [27]. The most favourable extraction methods could be short-duration and dark conditions. Accelerate solvent extraction (ASE) is such a method that extracts the chemical constituents from plant cells in inert conditions under high pressure within a short period. The extraction solvents also play a major role in anthocyanin yield and its quality. As it is soluble in polar solvents, methanol has been used widely for extraction. Studies have shown that methanol is widely used as a solvent for anthocyanin extraction. However, water, which is a low-cost and safe solvent, has also been found to have the potential to extract anthocyanins. To achieve higher extraction efficiency and yield of anthocyanins, acidic media, such as hydrochloric acid, formic acid, acetic acid, and phosphoric acid, are commonly used. The reason behind this is that the acids lower pH, which prevents the degradation of anthocyanin during the extraction process.

The most widely used method for determining the characteristics of anthocyanins, their abundance, and the ratios of different anthocyanidins is liquid chromatography-mass spectroscopy (LC-MS). The chemical characterization of anthocyanins is significant for the development of new bioinspired materials with specific biomedicinal values. The anthocyanin profile of blueberry [28], barberry [29], mulberry [30], cabbage [31], raspberry, grape skin, plum, and pomegranate were characterized by using mass spectroscopy techniques. Studies have been focused on the extraction and characterization of anthocyanins from plant by-products, which supports waste valorization and the value addition of food products. Plant by-products such as grape skin, fuji apple peel, red apple peel, and skin have been studied for their anthocyanin profile [6]. However, the anthocyanin profile of banana inflorescence has not been carried out for Cavendish banana inflorescence. Using plant by-products to extract medicinal compounds shows a bio-inspired strategy that could support sustainable production methods in bionics-related industries, such as the production of bio-compatible materials with antioxidant properties for stability.

This study is designed to carry out the extraction of anthocyanins from Cavendish banana inflorescence, dried under two drying conditions: freeze-drying and oven-drying. The extraction was carried out using ASE, with acidic water and methanol. The anthocyanin profile was characterized by LC-MS. The objective of this study includes (a) investigating the anthocyanin retention capacity of oven-dried and freeze-dried banana inflorescence, (b) determining the optimum conditions for extracting anthocyanins from banana inflorescence using ASE, and (c) characterizing the anthocyanins of banana inflorescence using mass-spectroscopy. In addition, this study will find the different colour characteristics of anthocyanins extracted via different drying methods and different extraction solvents.

## 2. Materials and Methods

### 2.1. Chemicals

The analytical grade chemicals were purchased from Sigma Aldrich, Sydney, Australia. The HPLC grade (≥99%), methanol, HCl, potassium chloride, Folin–Ciocalteau reagent, sodium acetate, DPPH, gallic acid, cyanidin-3-O-glucoside, and sodium carbonate were used for this study. Celite 577 (diatomaceous earth) was purchased from World Minerals, Santa Barbara, CA, USA.

#### Sample Collection and Preparation

Fresh banana inflorescences (FR) were sourced from banana farms in Wamuran, Queensland, Australia. Inflorescences from banana bunches, after the last bunch of fruits had opened, were carefully chosen for this study. This is also the typical stage for debelling on farms. To ensure minimal heat stress and the degradation of bioactive compounds, the debelling process took place in the evening. The inflorescences were promptly shielded with black polythene and transported to the laboratory within 24 h. Subsequently, the inflorescences underwent a thorough cleaning process, followed by the removal of the outer discoloured bracts extensively exposed to the sun. For this study, both bracts and male flowers were used together as they naturally assembled. The cleaned inflorescence was divided into three equal-sized sections, and only the middle section was further processed. The selected middle section was then carefully cut into approximately 0.5 cm × 0.5 cm small pieces using a sharp knife. These pre-cut samples were divided into two groups for oven-dried and freeze-dried extractions. Finally, the samples were meticulously wrapped in aluminums foil and stored in airtight sealer bags in a freezer at −20 °C for subsequent experiments.

**Figure 4:**
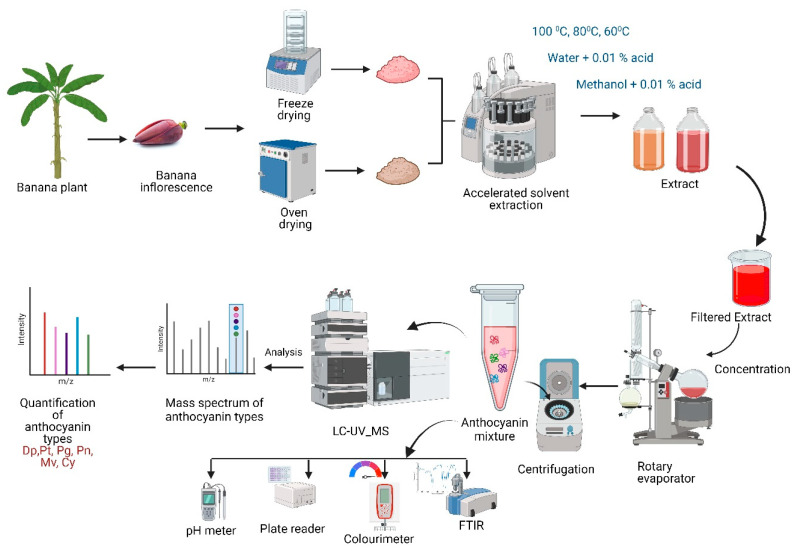
Method of extraction, isolation, and identification of anthocyanin profile for banana inflorescence.

The experimental design is illustrated in Figure 4. This study aims to investigate the anthocyanin profile of the banana inflorescence that was dried using both freeze-drying and convective oven-drying methods. Fresh banana inflorescence was dried under two drying conditions: in a convective oven and a freeze-dryer. The dried banana inflorescence was ground into a powder. The freeze-dried and oven-dried powder were extracted using the accelerated solvent extraction (ASE) method. The extracted solvents were filtered and further purified via centrifugation using a laboratory centrifuge, and the supernatant was taken for the analysis. The extracted solvent was then subjected to a UV-vis analysis for TPC, TAC, and DPPH. The purified anthocyanin extract was then placed in the LC_MS for profiling and quantification. The quantification was carried out using the standard curve prepared with cyanidin-3-O-glucoside. The extracted banana inflorescence was analyzed for its colours, total anthocyanin content, total phenolic content, total antioxidant activity, and pH sensitivity.

### 2.2. Processing Samples

#### 2.2.1. Oven-Drying

For this experiment, we utilized a convective oven (Labtek ODWF24, Grand Rapids, MI, USA). The banana inflorescence, which had been cut into small pieces, was evenly spread on metal trays (lined with aluminium foil) to form a fine layer. These trays were then placed in the convective oven, and the temperature was set to 55 °C, following the method outlined by Schmidt et al. [32]. After 24 h, the samples were stored in a dark, dry location within sealed bags then wrapped in aluminium foil until further processing.

#### 2.2.2. Freeze-Drying

For freeze-drying, the SP Scientific Benchtop Pro Freezer was utilized. Samples were pre-frozen in a freezer at −80 °C for 4 to 6 h before being they were placed in the freeze dryer. The samples were transferred to the freeze-drying chamber once the temperature reached −80 °C and the pressure dropped to −200 µB. It took 7 days to completely remove the moisture from 500 g of banana inflorescence. The freeze-dried banana inflorescence was then stored in a dark, dry place in sealed bags and covered with aluminium foil until milling was conducted.

#### 2.2.3. Milling

The Retsch miller fitted with a 2 mm sieve was used to grind the oven-dried and freeze-dried banana inflorescence into uniform fine powder to facilitate efficient extraction. The milled powder was stored in a dark sealed container for extraction within one week.

#### 2.2.4. Extraction

The extraction of bioactive compounds from banana inflorescence was performed using a green method known as accelerated solvent extraction (ASE), also referred to as pressurized liquid extraction (PLE). While the ASE method has been extensively studied for food products rich in bioactive compounds, there is no available literature on its application to banana inflorescence. For this study, the ASE 350 Dionex extractor (Thermo Fisher Scientific, Waltham, MA, USA) was used, following the method described by Azian et al. [33], with some modifications.

The extraction protocol involved a pressure of 1500 psi, a 60 s purging time per cycle, and a flush volume of 60%. The extraction process consisted of three cycles, each lasting 7 min, with an additional 5 min for preheating. Stainless steel extraction cells with a capacity of 36 mL were used, and the bottom of each cell was lined with an ASE filter glass fibre (34-Thermo Scientific grade) before loading the samples. Two grammes of oven-dried and freeze-dried banana inflorescence were added to each cell. The cells were then filled to 75% capacity by adding 5.0 g of diatomaceous earth (DE) (Thermo Fisher Scientific, P/N 062819) and then thoroughly mixed with the dried banana inflorescence sample.

**Figure 5:**
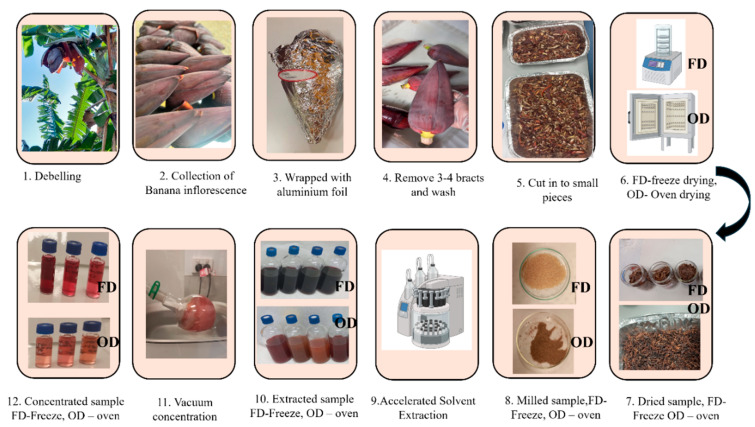
Process of extracting anthocyanin from banana inflorescence.

The extraction was performed using two solvents: acidified water and acidified methanol (methanol with 0.01% HCl), at temperatures of 60 °C, 80 °C, and 100 °C, as explained in Table 1. After extraction, the volume of the extract was adjusted to 200 mL with the same solvent used for extraction. The extracts were then filtered using sterile 0.22 μm syringe filters before being analyzed by LC-MS. The filtered extracts were stored in a refrigerator at −4 °C for 3 to 4 days before the analysis. These processing steps are explained in Figure 5.

The final extracts were used to determine the total antioxidant content, total phenolic content, and total anthocyanin content. These extracts were further examined to investigate their colour, pH sensitivity, and UV-Vis absorbance.

### 2.3. Physicochemical Characteristics

#### 2.3.1. Colour

The colour characteristics L, a*, b*, c* and h* values of the ABI were determined using the Konica Minolta Cr-10-plus colorimeter (Konica Minolta, Tokyo, Japan). Prior to measurement, the equipment was standardized using a white paper. The samples were then analyzed using a 1.0 cm path-length optical glass cell within the colorimeter. CIE values were measured in total transmission mode, with illuminant C and a 10 degree observer angle.

$$Chroma\left(C*\right)=\sqrt{{a*}^{2}+{b*}^{2}}$$

The hue angle (H°) describes the colour perception and the chroma (C*) describes the saturation of colour.

$$H^{{}^{\circ}}=\tan-1\frac{b*}{a*}$$

The colorimeter measures the brightness (L*) value and two coordinates: a* and b*. The A L* value of 100 represents absolute white, while a value of 0 represents absolute black. Positive a* (+a*) values indicate redness, while negative a* (−a*) values indicate greenness. Similarly, positive b* (+b*) values indicate yellowness, while negative b* (−b*) values indicate blueness.

The chroma (C*) value represents the intensity or saturation of the colour. The hue angle (h°) is characterized by the colour wheel, where specific angles correspond to different colours: 0° (red), 30° (orange), 60° (yellow), 90° (lime), 120° (green), 150° (turquoise), 180° (cyan or cobalt), 210° (blue or navy), 240° (violet), 270° (magenta), 300° (crimson), and 330° (pink).

#### 2.3.2. pH

pH values of the ABI samples were examined by using a Metler Toledo S210 digital pH metre (Metler Toledo, Columbus, OH, USA). The pH metre is calibrated before taking measurements.

#### 2.3.3. Total Soluble Solids (°Brix)

The total soluble solids of the ABI treatments were evaluated by refractometer using an RFM 342 Refractometer (Bellingham Stanley Ltd., Weilheim, Germany) and expressed in Brix values.

#### 2.3.4. FTIR Analysis of ABI

The anthocyanin characteristics of the ABI samples were examined as per the method explained by [34], with some modifications. An FTIR analysis, which was conducted using an infrared Diamond ATR-IR Nicolet iS50 (Thermo Scientific, USA) spectrometer (Nickolet FTIR), recorded the spectrum between 4000 and 375 cm^−1^, with a resolution of 1 cm^−1^ and the accumulation of 64 scans (scan rate 0.4 cm^−1^ s^−1^) at room temperature. The results were exported to an OMNIS software for analysis. All the ABI samples were compared with the standard cyanidin as a fingerprint for anthocyanin.

#### 2.3.5. UV-Vis Absorbance

The ABI samples were diluted with aqueous pH 1.0 and 4.5 buffers and the absorbance wavelength are recorded. The extracts were scanned on the UV-vis using a Synergy Biotek HTX multimode microplate reader (Biotek, Winooski, VT, USA) within the wavelength 300–750 nm to determine the absorbance range for pH 1 and pH 4.5.

#### 2.3.6. Total Phenolic Content

The total phenolic content was investigated using the Folin–Ciocalteu method. [35] with some modifications. Folin–Ciocalteu reagent (2 N) was used for this experiment. In brief, 790 µL of distilled water was added to 10 µL of the extract prepared in 1.5 mL Eppendorf tubes. Then, 100 µL of the Folin–Ciocalteu solution was added, and the tubes were left at room temperature for 5–8 min. After that, 150 µL of 20% sodium carbonate was added to each tube and mixed well. Then, the samples were incubated at room temperature (20 °C) in the dark for 2 h. After incubation, 200 µL of aliquots from each tube were placed on a 96-well microtiter plate, and finally, the absorbance was read at 765 nm using a Synergy Biotek HTX multimode microplate reader. The samples were compared against a gallic acid standard curve, and the results were expressed as a gallic acid equivalent (mg GAE/g sample).

#### 2.3.7. Total Antioxidant Activity

The total antioxidant activity of banana inflorescence was assessed using the 2,2-diphenyl-1-picrylhydrazyl (DPPH) free radical scavenging activity method explained by Yu [36], with some modifications. In brief, 300 µL of the DPPH solution was combined with 300 µL of the sample/standard/blank, followed by a 30 min incubation at room temperature (27 °C) in the dark. Subsequently, the absorbance was measured at 517 nm using a Synergy Biotek HTX multimode microplate reader with Gen 5.3 software. The absorbance results were then converted into antioxidant activity using a standard curve of ascorbic acid, and the results were expressed as µmol of ascorbic acid equivalents. The DPPH scavenging activity was calculated using the following equation.

$$\%\;\mathrm{DPPH}\;\mathrm{radical}\;\mathrm{scavenging}\;\mathrm{activity}=\left[\frac{\left(A_{0}-A_{1}\right)}{A_{0}}\right]\times 100$$

where A_0_ is the absorbance of the control without a sample (Blank) and A_1_ is the absorbance of the sample.

#### 2.3.8. Total Monomeric Anthocyanin Content

The total anthocyanin content was determined using the pH differential method, following the AOAC Official Method 2005.02. First, the extracted samples were diluted with two buffers: a 0.025 M potassium chloride buffer at pH 1 and a 0.4 M sodium acetate buffer at pH 4.5. Then, the absorbance of the samples was read using a UV-Vis spectrophotometer (Synergy Biotek HTX multimode microplate reader) with Gen 5.3 software at two specific wavelengths: 520 nm and 700 nm. Finally, the total anthocyanin content was calculated as cyanidin-3-glucoside equivalents using the following equation.

$$\begin{array}{c} C=\frac{A\times Mw\times Df\times 10^{3}}{\varepsilon \times l}\\ (\mathrm{Absorbance})=[\left(A\;520-A\;700\right)\;pH1.0-(A\;520-A\;700)\;pH4.5 \end{array}$$

where A is absorbance; Mw = molecular weight (448.8 g/mol for cyanidin-3-glucoside); Df = dilution factor; 10^3^ = conversion from gram to milligram; ε = molar extinction coefficient; L × mol^–1^ × cm^–1^ (26,900 L/mol/cm for cyanidin-3-glucoside); and l = pathlength (1 cm).

#### 2.3.9. Characterization of Anthocyanins by LC-MS Analysis

In the investigation of the anthocyanin profile, the method outlined by [32,33,35] was followed, with some alterations. In summary, LC-UV–MS was used to characterize the anthocyanins in banana inflorescence. A highly sensitive and high-resolution Shimadzu LCMS-8050 triple quadrupole instrument in the MRM mode with a PDA detector and a reverse phase column (Kinetex EVO C18, 100 mm × 2.1 mm × 2.6 μm) was employed. The PDA detector operated at a wavelength range of 500 nm–600 nm, with a spectrum resolution of 256. Mobile phase A consisted of 10% (*v*/*v*) formic acid in water, while mobile phase B was 10% formic acid in a 60:40 (*v*/*v*) mixture of methanol and acetonitrile. Blank (solvent) samples and standards were prepared using LCMS grade water, filtered through a 0.22 µM filter, and then loaded into inserts before being injected into the instrument, similar to the filtered banana inflorescence extracts. For absolute quantification, cyanidin-3-O-glucoside at various concentrations (50, 25, 12.5, 6.25, 3.125, and 1.56 µg/mL) was utilized to establish the calibration curve. Five microliters of banana extract were injected at an average flow rate of 0.4 mL/min for 10 min at 40 °C and 4800 psi. The MS investigation was conducted in automated mode, and machine control, data acquisition, and analyses were performed using Skyline software.

#### 2.3.10. pH Sensitivity and Colour Change

The pH differential method was used to evaluate the colorimetric indicator’s response to changes in pH. Buffer solutions with varying pH values were used to assess the indicator’s pH sensitivity. The pH sensitivity of extracted anthocyanin was studied using the methods explained by Yan et al. [37] and Zanoni et al. [31], with some alterations. 2 mL of the extracted anthocyanin-rich banana inflorescence were added to pH 1–14 buffer solutions (0.1 mol/L) for 5 min at room temperature. Then, the colour change was recorded using the colorimeter (Konica Minolta Cr-10-plus-Japan).

### 2.4. Statistical Analysis

The statistical differences were determined using Minitab software. The analysis of variance (ANOVA) test and the Turkey’s test were conducted to determine the significant difference. If *p* < 0.05 was considered as significantly different. Data were reported as the average ± standard deviation. All experiments were conducted in triplicates.

## 3. Results

The physicochemical properties of extracted anthocyanin from freeze-dried and oven-dried banana inflorescence at different extraction conditions were analyzed to determine the optimum extraction conditions.

### 3.1. Physicochemical Properties

The colour properties, pH, Brix value, total phenolic content (TPC), total antioxidant activity %, and total anthocyanin content (TAC) of different treatments were summarized in Table 2.

#### 3.1.1. Colour

The colour analysis data are presented in Table 2. The colour parameters a*, C*, and h* values differed significantly (*p* < 0.05) among treatments. All the extracts extracted from FD and OD samples exhibited better colour retention and higher redness values compared to the fresh extracts FRW and FRM control samples. The redness values ranged between 1.43 ± 0.05 and 38.09 ± 0.16. The higher a* value, indicating high redness properties, resulted in the descending order as FDM > FDW > ODM > ODW. The FDM samples exhibited the redness values in the order of FDM2 > FDM1 > FDM3. The FDM samples even had a concentrated dark maroon–red colour, indicating a high saturation of the dark red colour and less yellow colour. The colour gradually enhanced during the extraction at high temperatures from 60 °C to 80 °C, but the redness properties dropped sharply after 80 °C. The highest reported redness value is 38.09 ± 0.16 at 80 °C.

Chroma is another significant parameter in determining colour properties. The chroma values were calculated and summarized in Table 2 for ABI. The chroma values ranged between 7.27 ± 0.05 to 40.02 ± 0.13. The highest chroma values resulted in FDM2, followed by FDW2, while the lowest was reported as FRW, which is the control sample. This finding further confirms the vibrant chroma properties of the ABI extracted at 80 °C. Both water and methanolic extracts have shown their superior chroma values at 80 °C compared to 60 °C, reflecting the increasing colour at elevated temperatures. A similar relationship has been concluded in previous studies on mangosteen pericarp [21]. However, at 100 °C, a steady decline of chroma values was observed, which could be due to the oxidation of pigments responsible for red colour at extreme temperatures.

The hue values refer to the angle from redness, and hue values closer to 0 are considered high in redness. What is striking in this table is that all the treatments showed a hue value below 1, except the control treatment. Similar hue values were reported for mangosteen pericarps, suggesting 0–30° as a redness parameter [21]. Current findings can further confirm that banana inflorescence had better colour characteristics, specifically redness values, which are important for food industries as a natural pigment.

According to the results, colour properties reported for ABI are dominated by FDM, giving higher redness, high chroma, and high hue values. These properties are highly important for determining the applications of anthocyanin extracts for innovative and smart product development. Overall, we can conclude that ABI is a highly vibrant red colour extract but, the colour properties of ABI are highly dependent on the drying method and extraction parameters.

#### 3.1.2. pH

The pH values for ABI are presented in Table 2. There was a significant difference in pH values among the treatments (*p* < 0.05). The pH values ranged from 6.76 ± 0.05 to 7.76 ± 0.05. The control samples had pH values of 7.76 for FRW and 7.33 for FRM. Higher pH values were observed for ABI from ODM and ODW samples. These treatments resulted in poor colour retention for a*, h*, and c* values, as discussed in Section 3.1.1. This observation suggests that the decrease in anthocyanin concentration at higher pH levels led to lower redness values. The FDM treatments, which had higher colour retention properties, varied between 5.33 ± 0.05 to 5.76 ± 0.05. Similar pH ranges were reported for red cabbage, which ranged between 5.11 and 5.35 [38] whereas 5.36 pH had the highest AOC and Brix values.

#### 3.1.3. Brix

The Brix values of ABI are presented in Table 2. The *p*-value was reported as 0.371, as there is no significant difference among treatments. All the treatments showed higher brix values compared to the control samples. However, there is a slight difference among treatments, which is noticeable. The treatments that had lower pH showed higher Brix values as FDM values range between 18.30 and 18.46. The lowest values were reported from ODW treatments. The reported brix values showed that all the selected BI samples are in the maturity stage, as the brix value is closer to 20. According to the previous literature, lower brix values were reported from fruits and vegetables before the ripening stage, which is not suitable for consumer attraction. Therefore, we can conclude that the BI samples chosen for this study were in the correct maturity stage, and it will indirectly support the sensorial properties of products.

#### 3.1.4. FTIR Analysis

**Figure 6:**
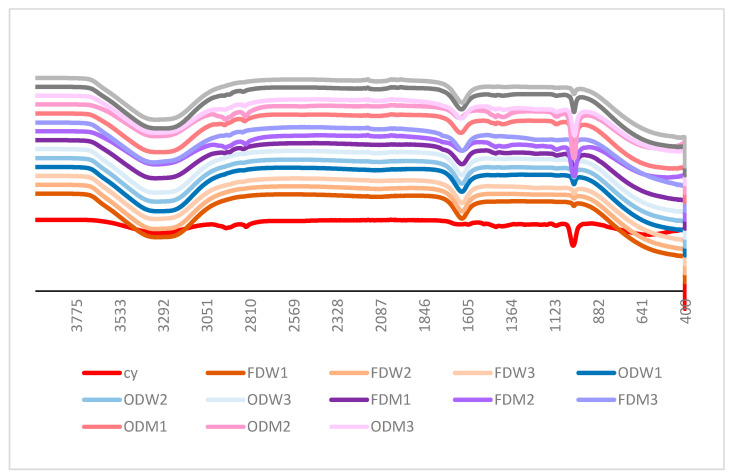
FTIR analysis for ABI-*cy-Cyanidin standard (1 mg/mL).

The FTIR analysis for anthocyanin identification (ABI) is illustrated in Figure 6. The most promising bands related to anthocyanins were observed in the range of 1600 cm^−1^ to 1000 cm^−1^. The cyanidin (CY) standard sample exhibited several characteristic bands at 3309, 2940, 2830, 1538, 1478, 1445, 1277, 1171, 1106, and 1018 cm^−1^. Notably, the significant bands related to anthocyanins range from 1600 cm^−1^ to 1000 cm^−1^.

A prominent band at 3309 cm^−1^ can be attributed to C-H stretching, typically observed in the range of 3000–3100 cm^−1^, from sugar vibrations and phenolic O-H groups. Peaks near 1600 cm^−1^ represent functional groups associated with aromatic C=C stretching. The peaks at 1018, 1106, and 1171 cm^−1^ stretched within the 1000–1200 cm^−1^ range and are associated with C-O-C stretching in flavonoids, which are key components of anthocyanins. These findings are consistent with previous literature on the FTIR analysis of culinary banana inflorescence [34], which reported aromatic ring stretching at 1516, 1261, and 1072 cm^−1^. Furthermore, similar fingerprints were observed for anthocyanin extracted from maize [39]. All the tested samples exhibited similar bands compared to the CY standard sample, confirming the presence of anthocyanins. However, we observed that the oven-dried water (ODW) and freeze-dried water (FDW) extracted samples did not display any peaks at 2940 and 2830 cm^−1^ across all treatments. This absence could indicate that specific anthocyanidins are not present in water-extracted samples, which aligns with the colour analysis showing lighter, less red coloration for these samples.

In contrast, methanolic extracted samples demonstrated that FTIR bands are more closely aligned with the CY standard. Notably, FDM and ODM samples exhibited bands similar to cyanidin, albeit in lower quantities. This suggests that methanolic extraction is more effective in isolating anthocyanins, likely due to the solubility properties of the anthocyanidins in methanol. In summary, the FTIR analysis confirms the presence of anthocyanins in the samples, with methanolic extraction proving more effective than water extraction. The absence of specific bands in ODW and FDW samples suggests a lower anthocyanidin content, which is further supported by the colour analysis results. Our findings are consistent with previous studies, further validating the use of FTIR spectroscopy in anthocyanin identification.

#### 3.1.5. UV-Vis Absorbance

**Figure 7:**
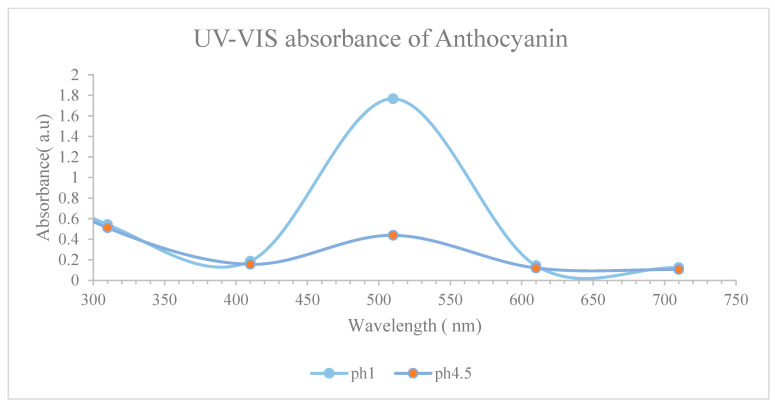
UV-Vis absorbance wavelength of anthocyanin at pH 1.0 and 4.5.

To determine the maximum absorbance spectrum, the extract was dissolved in distilled water and analyzed using a spectrometer with a wavelength range of 300 to 700 nm. Measurements were recorded for both pH 1 and pH 4.5 treatments. The results, shown in Figure 7, illustrate that the absorption peak occurred within the 500 to 530 nm range, consistent with the absorption spectrum of the anthocyanin colour group. Specifically, Figure 7 indicates that the maximum absorption point was at a wavelength of approximately 510 nm (λvis-max = 510 nm). A similar maximum absorbance wavelength was reported for *Carissa carandas* at 510 nm [23]. The general UV-VIS anthocyanin absorbance spectra also show similar peaks at 510 nm, as per our findings [40].

#### 3.1.6. Total Phenolic Content

The total phenolic content of ABI from FD and OD samples shows significant differences among samples, which are presented in Table 2. All the ABI showed a greater concentration of TPC compared to both control samples, FRW and FRM. This finding concludes that TPC has been enhanced during the drying process. This could be due to the concentration of dry matter content after evaporating water from the cells. Moreover, during the drying process, the cell walls break down and release the content from the vacuole to the extractant surface. A similar finding has been reported for mangosteen, which showed an increased TPC for both FD and OD mangosteen compared to fresh samples [21].

After conducting further statistical analyses of the data, we found that the FD samples had significantly higher concentrations of TPC compared to OD. The reported TPC values were approximately 45.17 ± 0.57, 42.5 ± 1.00, and 37.1 ± 0.51 for FDM1, FDM2, and FDM3, respectively. The lower TPC in OD samples may be due to the thermal degradation of phenolic compounds during longer exposure to heat.

Our findings are consistent with previous reports on higher TPC concentrations in freeze-dried black grape samples compared to OD samples [41]. To the best of our knowledge, this is the first study to compare drying methods, solvent concentrations, and extraction temperatures on the TPC of ABI.

The analysis of results for ODM and ODW samples found that the TPC concentration for ODM samples ranged from 27.83 ± 0.57 to 28.83 ± 0.57. The TPC concentration for ODW samples was significantly lower compared to ODM samples, showing a decline of 30–40%. This decrease was also observed in FDW and FDM samples. The decline in TPC could be due to the characteristics of methanol, which is a high-polar solvent and can efficiently extract phenolic compounds that are not soluble in water. Additionally, methanol has antioxidant properties that can prevent the oxidation of phenolic compounds.

The TPC results for ODW3 were similar to the reported TPC for Cavendish BI in another study, which was 16.9 mg/g [32]. However, it is important to note that the reported results for OD BI were from ethanolic extract, extracted under an ultrasonication bath, so comparing our results with previous studies is complicated due to differences in parameters such as the drying method, extraction solvent, and extraction temperatures used.

Furthermore, the extraction temperature of treatments showed a significant difference among treatments. It was observed that TPC slightly increased from 60 °C to 80 °C, possibly because the increase in temperature enhanced the extraction capacity. However, further increasing the temperature up to 100 °C decreased the TPC for almost all the samples. This led to the conclusion that 80 °C is the suitable temperature for conducting TPC extraction from banana inflorescence under the given drying and extraction parameters.

Overall, our experimental findings showed significantly higher concentrations of TPC for FDM, ODM, and FDW samples compared to TPC reported for BI in the literature. This can be explained by the results described by Ramírez-Bolaños et al. [42], concluding TPC as 1075.02 ± 20.20 mg/100 g and highlighting the non-extractable polyphenols percentage as 91%. Throughout the experiments, a range of extraction parameters were implemented to maximize the extraction process. These included utilizing acidic extraction, high-pressure extraction using ASE, conducting extraction under inert dark conditions using zirconium cells, minimizing extraction time, limiting exposure to light, and grinding dried material into microparticles. These parameters were instrumental in accelerating the phenolic extraction rate.

#### 3.1.7. Free Radical Scavenging Activity with 2,2-Diphenyl-1-Pricrylhydrazil (DPPH) Assay

The antioxidant activity of ABI was assessed using DPPH assays and summarized in Table 2. Bioactive compounds, such as anthocyanins, are abundant in ABI, providing excellent antioxidant activities. The results revealed that ABI is a rich source of antioxidants, which exhibited 63–82% scavenging activity for FDM and 60–62% for ODM. Overall, the treatments resulted in a higher concentration of DPPH compared to the control treatments, FRW and FRM. This result supports the idea that drying could enhance antioxidant activity. The DPPH % of extracts showed a phenomenally higher percentage for FDM than ODM. Statistical analyses confirmed that the drying process had a significant impact on the antioxidant capacity as well as their interaction. This finding is consistent with Nawawi et al.’s [21] findings on mangosteen, which demonstrated that drying affects antioxidant activity and total phenolic content.

Further examination of the results confirmed that there is a significant difference among extraction solvents used for extractions. The results showed that DPPH % for FDM ranged from 63 to 82% and, for FDW, it ranged from 28 to 30%. The DPPH % for ODM and ODW was 60–62% and 31–59%, respectively. These findings can conclude that methanol acts as a favourable solvent for extracting FD, and it has the potential to extract 50% more than OD. This finding can be supported by the literature. Methanol has been reported as a highly extractable solvent in previous research on S. buxifolia branches [43] and Scandinavian berry species [44], suggesting that methanol is the best solvent for bioactive compound extraction.

Another finding is that DPPH% showed varying results at different temperatures, similar to our previous findings for TPC. The statistical summary further confirmed that the temperature had a significant impact on the same samples with different extraction temperatures. The findings reported an increasing trend in DPPH% from 60 °C to 80 °C for FDW, ODW, FDM, and ODM samples. These findings are in line with the reported findings for rice berry bran with an increasing DPPH %, with temperatures from 60 °C to 80 °C [45]. However, the results showed a decline in DPPH % at the temperature of 100 °C, except in FDM samples. These relationships may be explained by the instability nature of antioxidants, such as anthocyanin, at elevated temperatures. Conversely, the FDM sample reported DPPH % in the order of 82.48% ± 0.01 > 71.33% ± 0.08 > 63.72% ± 0.22 for FDM1, FDM2, and FDM3, respectively. This contradictory finding might be explained by the antioxidant preservation ability of methanol, even at high temperatures.

#### 3.1.8. Total Monomeric Anthocyanin

Table 2 describes the total anthocyanin content for ABI samples. The results exhibited a significant difference among samples. This statistical difference concluded that the drying method, extraction solvent, and extraction temperature have an impact on the TAC of BI.

Our findings revealed that TAC for BI ranged from 15.58 ± 0.67 to 95.07 ± 3.5 mg/100 g. This variation occurred due to drying and extraction parameters. The TAC could be arranged in the order of FDM > FDW > ODM > ODW. The FDM2 reported a higher concentration of anthocyanin, which is 95.07 ± 3.5 mg/100 g. This finding is within the range of the TAC reported as 65–119 mg/100 g for Musa acuminata BI [9]. However, our results exhibited significantly higher values compared to the TAC reported as 57.29 mg/100 g [46]. The discrepancy could be attributed to many factors. This research was conducted by Begum and Deka [46], who have studied the Musa ABB cultivar. Genetic variance could cause variable TAC, as explained by another study [9]. Another possible reason could be the extraction parameters.

According to the findings, both OD and FD samples presented higher concentrations of TAC compared to fresh samples. This suggests that both drying methods enhanced the extractable anthocyanins. Our finding is contradictory to a previous report on black grapes, which described that oven-drying drastically reduced the anthocyanin content while freeze-drying had no loss compared to fresh black grapes [41]. However, a study conducted on mangosteen reported similar results, showing increased TAC in both OD and FD samples, compared to fresh [21]. This suggests that in some plant samples, drying processes may concentrate anthocyanin or make them more extractable, possibly due to cellular or structural changes that occur during drying.

The findings demonstrated a significant increase in anthocyanin in FDM and ODM samples compared to ODW and FDW samples. These findings can further support our TPC and DPPH results, which conclude that methanol is a suitable solvent for bioactive compound extraction. This is further evidenced by the colour analysis described in the previous section, demonstrating higher redness values related to anthocyanins found in FDM samples. Moreover, the study on black grapes also reported higher retention of anthocyanins in freeze-dried samples compared to oven-dried and sun-dried samples [41].

As can be seen from Table 2, the TAC is significantly affected by extraction temperature. Though both TAO and TPC showed a linear relationship with temperature, TAC showed a decline in concentration at elevated temperatures. The samples ODW, ODM, and FDW demonstrated a 60–70% decrease in TAC from 60 °C to 100 °C. What stands out in Table 2 is the rapid increase in TAC of FDM, with the temperature from 60 °C to 80 °C. However, a further increase in temperature up to 100 °C does not make any changes to TAC, which reflects the maximum extraction of TAC at 80 °C.

These findings could be used to conclude that water is not a favourable extraction solvent for anthocyanin extraction from OD samples. However, methanol is suitable for anthocyanin extraction from oven-dried samples at temperatures of 40 °C–60 °C. For FD samples, both water and methanol could be used as a solvent, although methanol reported significantly high concentrations. However, for FD samples, the best extraction temperature for water extraction is 60 °C while for methanol is 80 °C–100 °C.

From the results, it can be concluded that FDM2 is the optimal condition that could extract the highest concentration of anthocyanins. For ODW samples, the best extraction conditions are reported at 60 °C.

#### 3.1.9. Characterization of Anthocyanins

The ODM, FDM, FRM, ODW, FDW, and FRW samples with the highest retention of anthocyanins were subjected to an HPLC analysis. Six different samples were examined for their anthocyanin profile.

**Figure 8:**
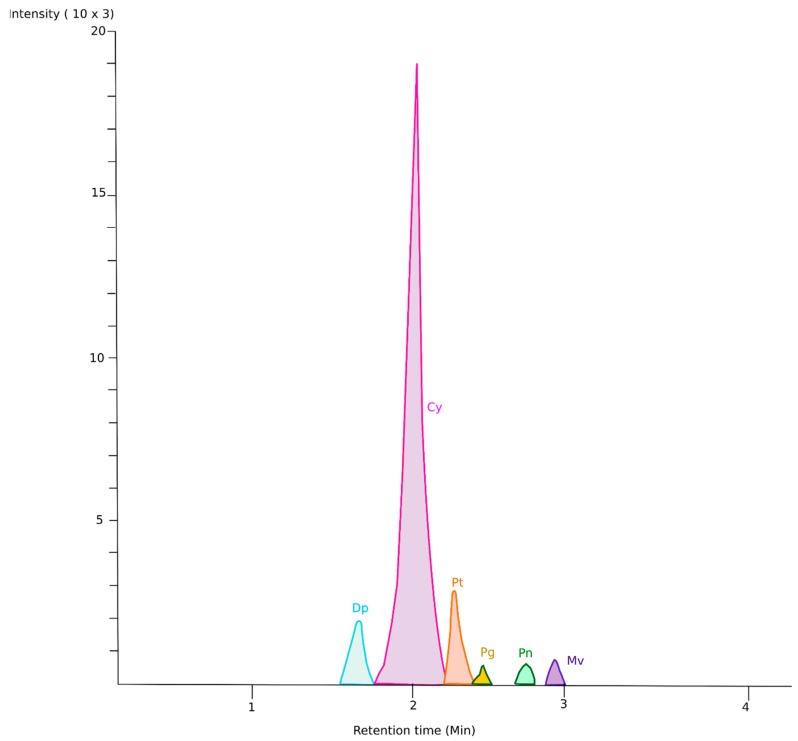
HPLC chromatogram of anthocyanin extracted from banana inflorescence.

The HPLC investigation for anthocyanin derived from banana inflorescence revealed six major peaks in its profile. Similar peaks were reported in the literature for many fruits and vegetables rich in anthocyanins. The order in which the anthocyanins were eluted in this investigation is similar to other studies on anthocyanins detection from plants [9,13]. According to Figure 8, we can see a prominent peak eluted as the second peak. The peaks were identified and listed in Table 3 by comparing the m/z, sequence of elution, and the reported literature on anthocyanin.

The main anthocyanin chemical is called flavylium (2-phenylbenzopyrilium) cation. The unique chemical structures and key positions (R1 and R2) on the benzene rings of flavylium cation result in distinct anthocyanin structures. An analysis reveals that ABI consists of six different anthocyanidins: delphinidin (Dp), cyanidin (Cy), petunidin (Pt), pelargonidin (Pg), peonidin (Pn), and malvidin (Mv). All these compounds are associated with the rutinosyl group. These compounds are responsible for various colours [47] due to their chemical characteristics (R1, R2 groups), which are listed in Table 3. The chemical structures of these compounds are illustrated in Figure 1.

The most abundant anthocyanidin, Cy, is an important compound with antioxidant, antidiabetic, anti-inflammatory, and cytoprotective effects [48]. Several studies proved that cyanidin acts as a strong natural chemical in fighting against prostate and bladder cancer [18]. Dp also offers promising health benefits such as antioxidative, anti-inflammatory, neuroprotective, cardioprotective, osteoprotective, and retinoprotective activities. Mv also exhibits pharmacological benefits such as cancer protection, as well as antioxidant and anti-inflammatory properties. Pt plays an important role in the defence mechanisms of chronic diseases due to its anti-inflammatory, anticancer, antidiabetic, anti-obesity, antioxidant, anti-osteoporosis, and photoprotective functions [49]. Peonidin has also shown antioxidant and chemoprotective effects [50]. Pg has the potential to induce antitumor cells, antimicrobial activities, antiplatelet activities, and in the treatment of hypoglycemia, retinopathy, and skeletal myopathy.

A similar study was conducted by Kerio et al. [13] for black and green tea. The anthocyanins were extracted using acidified methanol and preserved anthocyanin. The extracted anthocyanins were characterized by HPLC. The results demonstrated the availability of five different anthocyanins, namely Dp, Cy, pt, Pg, and Mv. Among these, malvidin (Mv) showed the highest concentration of anthocyanidin in both black and green tea. In another study, it was found that 80% of the anthocyanins in black carrots were retained when extracted using citric acid, resulting in high antioxidant activity and 90% colour retention [12]. These results are in line with our findings for BI, as acidified extract shows better preservation of anthocyanins.

**Figure 9:**
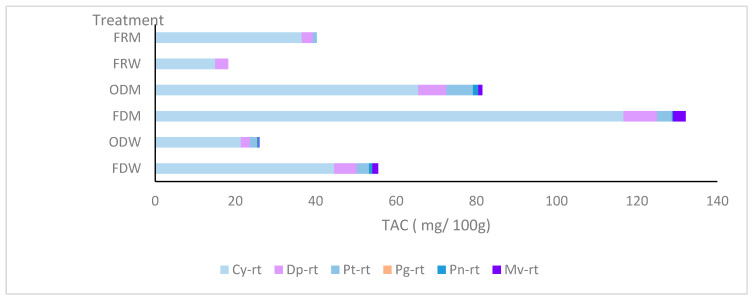
Different anthocyanins available in different extracts as a percentage.

The qualitative analysis of the results indicates that all treatments are consistent with the presence of anthocyanidins Cy-Rt and Dp-Rt. The findings suggest that Cy-Rt is the dominant anthocyanidin present in ABI, accounting for 80–90% of total anthocyanins, as illustrated in Figure 9. Dp-Rt is the second-highest anthocyanidin identified in ABI, ranging between 6 and 18%. Pt-Rt is eluted in methanolic extracts by 1–2% and water extract by 5–6%. Pn-Rt and Mv-Rt showed their presence on both OD and FD samples. However, their percentage is below 3%. Pg-Rt is only identified in fresh extracts, which is below 1%.

The results for the presence of different anthocyanidins varied depending on the treatment used. FRW only contained the main two anthocyanidins, Cy-Rt and Dp-Rt. On the other hand, FRM showed its ability to retain Dp-Rt, Cy-Rt, and Pt-Rt. Both OD and FD treatments exhibited a wide range of anthocyanidin retention compared to FRW and FRM. This could be attributed to the effective extraction of anthocyanins during the drying process, which enhanced the retention of anthocyanins compared to fresh samples. Pg-Rt was found in very small quantities only in FDW and ODW samples, indicating a limited extraction potential in methanol for extracting Pg. This could be due to the conversion of Pg into another compound during methanolic extraction. FDM and ODM samples did not contain Pg, which was only present in samples extracted in water. This may explain the lower redness values in water extracts, as pelargonidin is responsible for the orange colour [47].

The quantitative analysis of the results indicates higher concentrations for all treatments compared to spectrophotometric pH difference analytical methods. This could be due to the sensitivity of the HPLC instrument. Similar findings have been reported previously, showing significantly higher results for anthocyanins in tea compared to spectrophotometer analysis [13]. The higher concentration of anthocyanin identification by the HPLC method is further explained by Lee et al. [15], showing compatibility with our findings. After thorough examination, we have determined that the HPLC method is more precise as it can detect both anthocyanidins and anthocyanins. In contrast, the pH-differential method primarily measures less stable anthocyanidins, as it is based on the measurement of the flavylium cation. The HPLC assays successfully identified the presence of anthocyanins in the BI samples, as well as the presence of anthocyanins combined with rutinosides. This led to higher levels of total anthocyanins compared to the pH-differential method.

The highest concentration of anthocyanin is reported in the FDM sample at 130.01 ± 3.00 mg/100 g. This further supports our results for colour analysis, TPC, TAO, and TAC. These results confirm that freeze-drying is the most suitable method for sample processing, and methanol is the most favourable solvent for anthocyanin extraction. The lowest concentration was reported in the FRW sample, indicating that analyzing fresh samples using water as a solvent is not efficient. All treatments using methanol showed 2.5–3 times higher concentrations, indicating that methanol is a suitable solvent for anthocyanin extraction.

#### 3.1.10. pH Sensitivity and Colour Change

**Figure 10:**
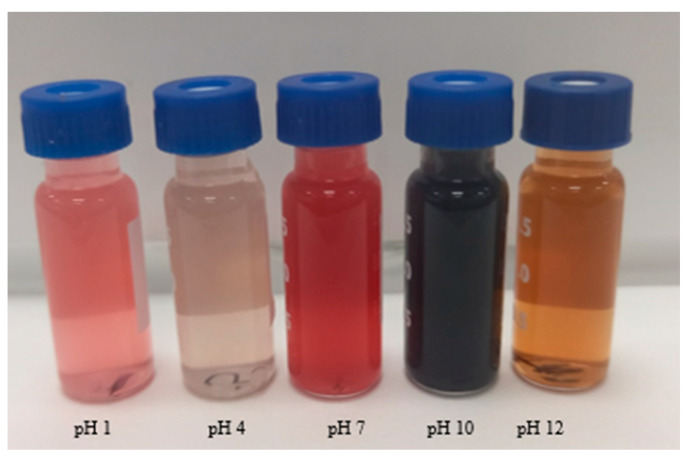
Response of anthocyanin extract at different pH.

Anthocyanins can change into different chemical structures and colours at different pH conditions [31]. Colorimetric results have shown that the colours of anthocyanin extracted from banana inflorescence range from pink to brown depending on the pH level, as illustrated in Figure 10. The observed colours at different pH ranges are as follows: pH 1–3 (pink), 4–6 (ash), 7–9 (red), 10–11 (dark purple–blue), and 12–14 (brown). The sensitivity of anthocyanin to pH is linked to the change in colour from flavylium cation [14] at lower pH to chalcone compounds (at higher pH). The light pink colour at pH 1 is caused by flavylium cation [37]. At pH 4–6, the colour changes to ash due to the carbinol pseudobase. A dark red colour at pH 7 is produced from an anionic quinoidal base. Above pH 10, brown-coloured anthocyanins can be observed due to structural changes to the chalcone. Previous studies have reported colour changes in anthocyanins from flavylium to the quinonoidal base in extracts from red cabbage, jamun, roselle, blueberry, black bean, grape skin, and purple sweet potato [51].

pH sensitivity of anthocyanin extracted from banana inflorescence shows its potential to be used as an indicator of intelligent film synthesis. These intelligent films can be used for many food and pharmaceutical applications, including microbial growth indicators and food spoilage indicators. Some studies show the application of films produced from anthocyanin. A recent study shows the potential of banana bracts anthocyanin to form a pH indicator film and its potential to be used as an indicator of food spoilage [27]. Similar intelligent products were made from anthocyanin extracted from red barberry [29] and purple-fleshed sweet potato [52].

As per our findings, we can conclude that anthocyanin extracted from Cavendish banana inflorescence shows contrasting colour changes in varying pH, which could be a source of pH indicators for intelligent product development.

## 4. Conclusions

In conclusion, this study has revealed the significant potential of Cavendish banana inflorescence as a natural source of anthocyanins, highlighting its importance in both industrial and health-related applications. The findings demonstrate that anthocyanins can be effectively preserved through optimal drying methods, with freeze-drying yielding the highest preservation levels. This research emphasizes the impact of various drying methods and extraction parameters on the concentration and bioactive properties of anthocyanins, showcasing their antioxidant and antimicrobial benefits, as well as the colour properties that make banana inflorescence a valuable resource for the pharmaceutical industry. Moreover, this study determined the different anthocyanidins available in banana inflorescence using HPLC methods and concluded that banana inflorescence is a rich source of cyanidin, malvidin, petunidin, pelargonidin and peonidin.

Furthermore, the unique colour-sensing properties of anthocyanins reveal the potential for their use as natural food colourants, providing a sustainable alternative to synthetic options without compromising human health. The study further suggests innovative applications of anthocyanins in intelligent food packaging, health indicators, and pH sensitivity due to their dynamic colour changes. However, the chemical instability of anthocyanins presents a challenge for industrial applications, necessitating further research aimed at mitigating degradation during processing. Understanding these factors is essential for developing anthocyanin-specific fingerprints and models, which could enhance their utilization across various industries.

Overall, the insights gained from this study provide a strong foundation for future research and development, paving the way for the incorporation of anthocyanins extracted from banana inflorescence into food, nutraceuticals, and pharmaceutical products, ultimately contributing to a more sustainable and health-conscious approach in these sectors.

## Figures and Tables

**Table 1:** Different combinations of drying methods, extracted solvent, and extraction temperatures.

Treatment	Drying Method	Extraction Solvent	Extraction Temperature (°C)
FDW1	Freeze-drying	Water	100
FDW2	Freeze-drying	Water	80
FDW3	Freeze-drying	Water	60
ODW1	Oven-drying	Water	100
ODW2	Oven-drying	Water	80
ODW3	Oven-drying	Water	60
FDM1	Freeze-drying	Methanol	100
FDM2	Freeze-drying	Methanol	80
FDM3	Freeze-drying	Methanol	60
ODM1	Oven-drying	Methanol	100
ODM2	Oven-drying	Methanol	80
ODM3	Oven-drying	Methanol	60
FRW	Fresh	Water	100
FRM	Fresh	Methanol	100

**Table 2:** Physicochemical properties of anthocyanin extracted from banana inflorescence.

Treatment	a*-Redness	C*-Chroma	h*-Hue Angle	pH	Brix°	TPC (mg/g)	TAO %	TAC mg/100 g
FDW1 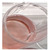	7.86 ± 0.05 k	11.75 ± 0.07 i	0.83 ± 0 b	6.76 ± 0.05 c	17.91 ± 0.01	28.16 ± 1.52 c,d	34.51 ± 0.44 i	32.34 ± 2.43 f
FDW2 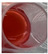	25.73 ± 0.11 b	32.88 ± 0.07 b	0.67 ± 0 e	6.2 ± 0.1 f	12.66 ± 9.78	30.17 ± 1.15 c	58.25 ± 0.25 f	33.5 ± 1.78 e,f
FDW3 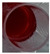	12.56 ± 0.05 h	14.23 ± 0.24 g	0.48 ± 0.03 h	5.86 ± 0.05 g	18.3 ± 0.07	28.83 ± 2.08 c,d	47.34 ± 0.44 g	52.6 ± 0 c
ODW1 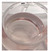	9.33 ± 0.05 j	12.42 ± 0.04 h,i	0.72 ± 0 d	6.66 ± 0.05 c,d	17.23 ± 0.03	13.83 ± 1.52 f	31.11 ± 1.27 j	15.58 ± 0.67 g
ODW2 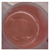	11.23 ± 0.05 h,i	16.23 ± 0.03 f	0.8 ± 0 b	6.53 ± 0.11 d,e	17.46 ± 0.04	19.5 ± 0.99 e	59.13 ± 0.51 e,f	16.36 ± 1.16 g
ODW3 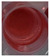	22 ± 1.73 c	25.64 ± 1.55 c	0.54 ± 0.02 g	6.16 ± 0.05 f	17.66 ± 0.03	16.83 ± 0.57 e,f	41.44 ± 0.25 h	18.7 ± 1.16 g
FDM1 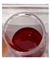	19.73± 0.11 d	20.5 ± 0.12 d	0.27 ± 0 j	5.56 ± 0.05 h	18.43 ± 0.03	45.17 ± 0.57 a	82.49 ± 0.01 a	92.92 ± 0.82 a
FDM2 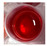	38.09 ± 0.16 a	40.02 ± 0.13 a	0.31 ± 0 j	5.76 ± 0.05 g,h	18.46 ± 0.02	42.5 ± 1 a	71.33 ± 0.08 b	95.07 ± 3.57 a
FDM3 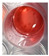	14.83 ± 0.05 g	16.29 ± 0.04 e,f	0.42 ± 0 i	5.33 ± 0.05 i	18.29 ± 0.09	37.17 ± 0.51 b	63.72 ± 0.22 c	70.91 ± 0.67 b
ODM1 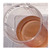	16.33 ± 0.11 f	21.04 ± 0.07 d	0.68 ± 0 d,e	7.33 ± 0.11 b	17.94 ± 0.01	27.83 ± 0.57 c,d	60.31 ± 0.25 d,e	30 ± 1.78 f
ODM2 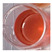	18.3 ± 0.17 e	25.29 ± 0.11 c	0.76 ± 0 c	6.36 ± 0.05 e,f	18.22 ± 0.03	28.83 ± 0.57 c,d	63.86 ± 0.67 c	45 ± 0.82 c,d
ODM3 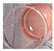	15.16 ± 0.05 f,g	17.55 ± 0.1 e	0.52 ± 0 g,h	6.46 ± 0.05 d,e	18.04 ± 0.04	28.81 ± 0.57 c,d	60.02 ± 0.25 d,e	40.32 ± 2.47 d,e
FRW 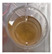	1.43 ± 0.05 l	7.27 ± 0.05 j	1.37 ± 0 a	7.76 ± 0.05 a	15.02 ± 0.03	17.17 ± 1.15 f	30.82 ± 1.11 j	12.46 ± 3.75 g
FRM 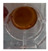	11.03 ± 0.05 i	13.35 ± 0.04 g,h	0.59 ± 0 f	7.33 ± 0.05 b	17.07 ± 0	26.5 ± 2.65 d	61.05 ± 0.44 d	34.48 ± 2.47 e,f

Values are the mean standard deviation, and those with different letters in the same column are significantly different (*p* < 0.05).

**Table 3:** Physicochemical chromatographic characteristics of anthocyanin pigments from Cavendish banana inflorescence.

Peak	Retention Time (min)	[M]^+^ *m*/*z*	Production ^+^ *m*/*z*	Identification	R1 Group	R2 Group	Responsible Colour
1	1.6	611.2	301.9, 463.9	Dp-3-rutinoside	OH	OH	Dark Purple
2	2.0	595.8	285.9, 447.9	Cy-3-rutinoside	OH	H	Red–Magenta–Purple
3	2.3	624.9	315.9	Pt-3-rutinoside	OH	OCH_3_	Blue–Purple
4	2.4	579.4	269.9	Pg-3-rutinoside	H	H	Orange–Dark Red
5	2.7	608.7	299.9	Pn-3-rutinoside	OCH_3_	H	Purple–Red–Orange
6	2.9	638.8	329.9	Mv-3-rutinoside	OCH_3_	OCH_3_	Dark Blue–Purple

## Data Availability

The original contributions presented in the study are included in the article, further inquiries can be directed to the corresponding author.
